# To unveil the causal relationship between immunophenotypes and colorectal cancer using two-sample bidirectional Mendelian randomization and mediation analyses

**DOI:** 10.1097/MD.0000000000049769

**Published:** 2026-07-10

**Authors:** Yi Zhang, Bingyuan Fei, Xuedong Fang, Suyan Tian

**Affiliations:** a Department of Gastrointestinal Colorectal Surgery, China-Japan Union Hospital of Jilin University, Changchun, Jilin, China; b Division of Clinical Research, The First Hospital of Jilin University, Changchun, Jilin, China.

**Keywords:** colorectal cancer, genome-wide association study (GWAS), immunity, mediator, Mendelian randomization (MR)

## Abstract

Colorectal cancer (CRC) is a leading cause of cancer-related death worldwide. The mechanisms underlying this trend are not yet fully understood. This study aimed to examine the potential role of genetically predicted immunophenotypes in the development of CRC. A two-sample bidirectional Mendelian randomization study was conducted to explore the relationship between 731 genetically predicted immune cells and CRC. Furthermore, a two-step Mendelian randomization approach was employed to assess the possible mediating effect of immune cells on CRC. The inverse-variance weighted method identified 5 immunophenotypes as significantly inversely associated with CRC risk: the odds ratios for CRC risk associated with activated CD4 regulatory T cells (%CD4 regulatory T cells), CD25++ CD45RA- CD4 nonregulatory T cells (%CD4 + T cells), CD25++ CD45RA- CD4 nonregulatory T cells (%T cells), CD25++ CD8 + T cells (%T cells), and CD64 + CD16 + monocytes were 0.925 (95% CI = 0.874–0.978, *P* = 6.516 × 10^−3^), 0.935 (95% CI = 0.878–0.995, *P* = .035), 0.936 (95% CI = 0.889–0.985, *P* = .011), 0.863 (95% CI = 0.786–0.948, *P* = 2.142 × 10^−3^), and 0.636 (95% CI = 0.519–0.778, *P* = 1.18 × 10^−5^), respectively. The mediation analysis indicated that the absolute count of CD25++ CD8 + T cells led to a 33.9% decrease in the risk associated with the percentage of activated CD4 regulatory T cells within CD4 regulatory T cells and CRC. Our analysis revealed that 5 immunophenotypes may be risk factors for CRC. Since other complementary methods have yielded inconsistent results, however, further investigation is necessary.

## 1. Introduction

Colorectal cancer (CRC) is one of the top 4 deadly cancers worldwide and causes nearly 900,000 deaths each year, accounting for about 10% of the global incidence and mortality rates of cancer.^[1,2]^ Early detection is essential for effective CRC treatment. The present treatment options for CRC include endoscopy, local surgical resection, radiotherapy, systemic treatments, ablation therapy, and palliative chemotherapy. Nonetheless, the efficacy of these treatments in advanced metastatic CRC is restricted.^[1]^ The understanding of CRC pathogenesis and potential mechanisms is still in its nascent stage, necessitating further research.^[1,3]^

The pathogenesis of CRC is multifaceted, with an unhealthy diet, obesity, and a sedentary lifestyle widely acknowledged as potential risk factors. Recent studies, however, have revealed a close association between peripheral blood immune cells and CRC onset.^[4,5]^ Immune cells are specialized cells that play critical roles in the body’s immune system, defending against infections, diseases, and foreign substances (such as pathogens like bacteria, viruses, fungi, and parasites). Immune cells can be broadly categorized into 2 main groups on the basis of their function and origin: innate immune cells and adaptive immune cells. They are integral to various inflammatory responses during cancer progression and treatment, playing a pivotal role.^[5–8]^ In CRC tumor tissues, these immune cells form an immune center that impacts the tumor microenvironment, influences the growth and spread of tumor cells, and thus possibly plays a role in CRC onset and progression as well.^[9–11]^

The origin of tumor-infiltrating Tregs remains ambiguous owing to their presence in both the bloodstream and resident tissues. The immune profile of a tumor is intricately linked to its tissue of origin, suggesting a potential relationship between tumor-infiltrating Tregs and tissue-resident Tregs. These findings indicate that Treg infiltration into tumors might be influenced by the tissue-specific immune microenvironment. Understanding the origin and dynamics of tumor-infiltrating Tregs is crucial for comprehending tumor-immune interactions and developing targeted immunotherapies.^[12,13]^ However, current CRC pathogenesis research relies primarily on observational cohort studies, which constrain the capacity to establish causality.

Mendelian randomization (MR) analysis uses genetic variants, which are fixed and randomly assigned at conception, as proxies to estimate the impact of an exposure on the outcome. MR is least subject to the influences of confounders and reverse causation.^[14,15]^ Currently, MR methods are rarely implemented to investigate the correlation between individual immune factors and the incidence of CRC. This study aimed to fill this gap by elucidating the underlying potential underlying mechanisms and eventually providing insights into how to optimize prevention and treatment strategies for CRC.

## 2. Materials and methods

### 2.1. Experimental data

Summary statistics from genome-wide association study (GWASs) for the inherited component of 731 immunophenotypes were obtained from the GWAS Catalog (https://www.ebi.ac.uk/gwas/), with accession numbers ranging from GCST0001391 to GCST0002121.^[16]^ These phenotypes include 539 immune traits, comprising 118 absolute cell counts, 389 mean fluorescence intensities of surface antigens, and 32 morphological parameters, in addition to 192 relative counts (ratios between cell levels). The data were gathered from a general population cohort of 3757 Sardinians as part of the SardiNIA project. This longitudinal study involved 6602 individuals native to central-eastern Sardinia, Italy, with a gender distribution of 57% females and 43% males, aged between 18 and 102 years. All participants underwent extensive genetic characterization, and 3757 were profiled for their immune response. The GWAS study tested 20,143,392 single-nucleotide polymorphisms (SNPs) and 1688,858 indels, genotyped using high-density arrays or imputed via a Sardinian sequence-based reference panel comprising 3514 individuals.

Data on malignant neoplasms of the colon and rectum, as classified by the International Statistical Classification of Diseases and Related Health Problems (ICD), were acquired from publicly available GWAS summary data in the Open GWAS project (ebi-a-GCST90018808 and ieu-b-4965) (https://gwas.mrcieu.ac.uk/). These 2 cohorts included 6581 cases and 463,421 controls (discovery set) ^[17]^ and 5657 cases and 372,016 controls from the European population (validation set).

### 2.2. Ethics statement

Ethical approval and informed consent were not needed, as this study exclusively utilized publicly available summary GWAS data.

### 2.3. Instrumental variables

Three criteria were employed to select eligible SNPs as instrumental variables (IVs).^[18]^ First, SNPs with *P* < 5 × 10^−8^ were identified and considered significantly associated with the exposure of interest at the genome-wide level, which corresponds to the relevance assumption. Second, SNP clustering was executed on the basis of linkage disequilibrium removal, with a linkage disequilibrium threshold of *R*^2^ > 0.001 and a distance within 10,000 kb. Finally, to reduce bias from weak IVs, *F* statistic values were calculated for each SNP to assess the statistical strength of the IVs. SNPs with *F* < 10 were deemed weak instruments and excluded, ensuring minimal weak instrument biases.^[19–21]^ SNPs from various studies were standardized in terms of their effects, and palindromic SNPs (i.e., SNPs composed of a base and its complementary base) were deleted.

### 2.4. MR analyses

A comprehensive assessment of the causal influence of immunophenotypes on CRC was conducted using MR analysis. These methods included inverse-variance weighted (IVW), MR-Egger, weighted median, and weighted mode methods.^[22–25]^ These MR techniques each make distinct assumptions and offer specific advantages and disadvantages.

Briefly, the IVW method combines Wald ratio estimates from multiple SNPs via a meta-analytic approach, yielding a robust estimate of the causal effect of the exposure on the outcome, provided that all genetic variants are valid instruments. As the primary analysis in this study, IVW was chosen for its high statistical power when the 3 core IV assumptions (relevance, independence, and exclusion restriction) hold. To comprehensively assess this relationship, supplementary MR methods, including MR-Egger, weighted mode, and weighted median, were also applied. Notably, owing to the limited sample sizes of the exposure and outcome GWAS datasets, no multiple testing correction was performed, and a nominal *P*-value threshold of <.05 was considered statistically significant.

Statistical analyses were performed to verify the validity of IV assumptions, particularly regarding horizontal pleiotropy (which corresponds to both independence and exclusion restriction assumptions). Heterogeneity was assessed using Cochran’s *Q* statistic, with a significance threshold of 0.05, indicating notable heterogeneity.^[26]^ In the presence of heterogeneity among IVs, random-effect IVW instead of fixed-effect IVW was employed for effect estimation. Pleiotropy was evaluated using the intercept in MR-Egger regression. A nonsignificant MR-Egger intercept (*P* > .05) indicated no evidence of directional pleiotropy. Additionally, forest and funnel plots were used to confirm the lack of outlier effects and to demonstrate the robustness of the correlation and the absence of heterogeneity, respectively. A leave-one-out analysis was conducted to determine the influence of each SNP on the overall results.

Finally, we conducted a two-step MR analysis to assess potential mediation effects. The mediation model was specified as follows: c’= c–a × b, where c represents the total causal effect of the immunophenotypes on CRC risk, a denotes the causal effect of the exposure on the mediator, and b signifies the causal effect of the mediator on CRC risk; thus, a × b is the product of the effect of the immunophenotypes on the mediator and the effect of the mediator on CRC, indicating the mediating effect from the exposure to the outcome. Therefore, c’ represents the residual effect of immunophenotypes on CRC after accounting for mediation.^[27]^

All analyses were executed via the TwoSampleMR and MendelianRandomization packages in R Software, version 4.3.2 (https://www.R-project.org).

## 3. Results

### 3.1. Forward MR analyses

To explore the causal effects of immunophenotype on CRC incidence, a two-sample MR analysis was performed, and the IVW method was used for the primary analysis. The ebi-a-GCST90018808 dataset was utilized as the discovery dataset ^[17]^, while the UK Biobank dataset was employed for validation. No overlap between the exposure and outcome GWASs is expected. In the discovery analysis, we observed 42 suggestive immunophenotypes (unadjusted *P*-value <.05) using the IVW method, as specifically illustrated in Figure S1, Supplemental Digital Content 1.

**Figure 1.**
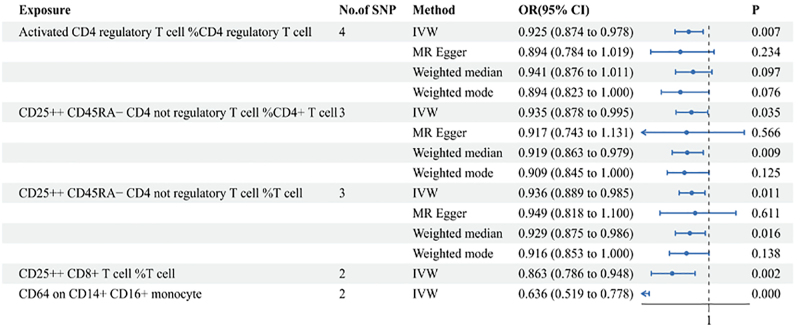
Forest plot of suggestive immunophenotypes to be causally related to colorectal cancer for the discovery dataset. CI = confidence interval, OR = odd ratio, IVW = inverse-variance weighted, SNPs = single-nucleotide polymorphisms.

**Figure 2.**
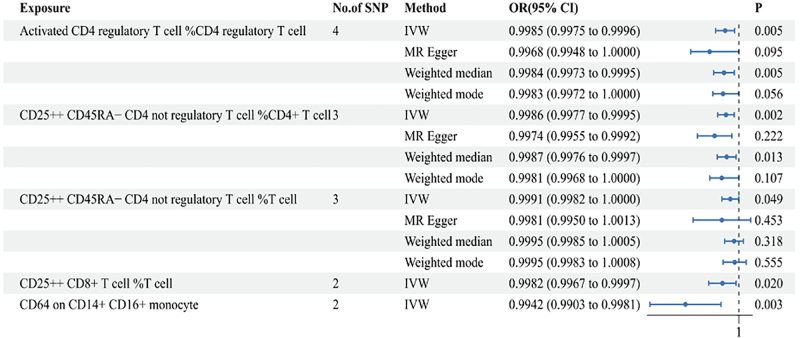
Forest plot of suggestive immunophenotypes to be causally related to colorectal cancer for the validation dataset. CI = confidence interval, OR = odd ratio, IVW = inverse-variance weighted, SNPs = single-nucleotide polymorphisms.

To validate the accuracy of the results, we subsequently utilized the UK Biobank dataset as a validation dataset for analysis and identified 5 immunophenotypes that reached significance (*P* < .05) in both datasets (Figs. 1 and 2). Five suggestive immunophenotypes were identified, 3 of which were within the Treg panel, one within the monocyte panel, and one within the TBNK panel.

In the discovery dataset, the IVW method estimated the odds ratios (ORs) for CRC risk associated with activated CD4 regulatory T cells (%CD4 regulatory T cells), CD25++ CD45RA- CD4 nonregulatory T cells (%CD4 + T cells), CD25++ CD45RA- CD4 nonregulatory T cells (%T cells), CD25++ CD8 + T cells (%T cells), and CD64 + CD16 + monocytes to be 0.925 (95% CI = 0.874–0.978, *P* = 6.516 × 10^−3^), 0.935 (95% CI = 0.878–0.995, *P* = .035), 0.936 (95% CI = 0.889–0.985, *P* = .011), 0.863 (95% CI = 0.786–0.948, *P* = 2.142 × 10^−3^), and 0.636 (95% CI = 0.519–0.778, *P* = 1.18 × 10^−5^), respectively. The ORs associated with activated CD4 regulatory T cells (%CD4 regulatory T cells), CD25++ CD45RA- CD4 nonregulatory T cells (%CD4 + T cells), and CD25++ CD45RA- CD4 nonregulatory T cells (%T cells) consistently showed a trend; however, these results were not significant when other MR methods were used.

In the validation dataset, the IVW method was used to estimate that the ORs for CRC risk were associated with the percentages of activated CD4 regulatory T cells, CD25++ CD45RA- CD4 nonregulatory T cells, and CD25++ CD45RA- CD4 nonregulatory T-cell %T cells; CD25++ CD8 + T-cell %T cells; and CD14 + CD16 + monocytes, 0.9985 (95% CI = 0.9975–0.9996, *P* = .005); 0.9986 (95% CI = 0.9977–0.9995, *P* = .002); 0.9991 (95% CI = 0.9982–1.0000, *P* = .049); 0.9982 (95% CI = 0.9967–0.9997, *P* = .020); and 0.9942 (95% CI = 0.9903–0.9981, *P* = .003). However, the results were not statistically significant when analyzed using the weighted mode, weighted median, or MR-Egger. Hence, these 5 immunophenotypes were deemed statistically significant only by IVW in both the discovery and validation data.

Heterogeneity (Cochran’s Q *P*-value >.05) or horizontal pleiotropy (MR–Egger intercept-derived *P*-value >.05) were not detected in all MR analyses (Table 1). Further investigation on scatter plots of MR methods and funnel plots also indicated no existence of heterogeneity or pleiotropy (Figure S2, Supplemental Digital Content 2 and Figure S3, Supplemental Digital Content 3 for funnel plots and scatter plots on the discovery set and validation set, respectively).

**Table 1:** Results of statistical tests on horizontal pleiotropy and heterogeneity.

Dataset	Immune traits	Cochran’s Q (IVW)		MR-Egger		
		*Q*	*P*-value	Intercept		*P*-value
Discovery data	Activated CD4 regulatory T cell %CD4 regulatory T cell (ebi-a-GCST90001487)	1.957	.581	0.041	.629	
Validation data		4.119	.249	0.002	.219	
Discovery data	CD25++ CD45RA- CD4 not regulatory T cell %CD4 + T cell (ebi-a-GCST90001511)	3.356	.187	0.021	.874	
Validation data		2.149	.341	0.001	.383	
Discovery data	CD25++ CD45RA- CD4 not regulatory T cell %T cell (ebi-a-GCST90001512)	0.762	.683	-0.013	.882	
Validation data		2.709	.258	0.001	.646	
Discovery data	CD25++ CD8 + T cell %T cell (ebi-a-GCST90001679)	0.003	.960	–	–	
Validation data		0.409	.522	–	–	
Discovery data	CD64 on CD14 + CD16 + monocyte (ebi-a-GCST90002011)	0.949	.330	–	–	
Validation data		1.638	.201	–	–	

### 3.2. Reverse MR analysis

Next, we conducted a reverse MR analysis, with CRC cells considered to be associated with the exposure and immunophenotype (including activated CD4 regulatory T cells, CD25++ CD45RA- CD4 nonregulatory T cells (%CD4 + T cells, CD25++ CD45RA- CD4 nonregulatory T cells, CD25++ CD8 + T cells, and CD64 + CD14 + CD16 + monocytes) as the outcome. The F statistics of all the SNPs were >10, indicating that there was no significant weak instrument bias. Briefly, reverse MR analysis provided no evidence indicating that the development of CRC may influence the activity of these 5 immunophenotypes. The MR–Egger intercept did not detect any horizontal pleiotropy, and the Q statistics revealed no heterogeneity. Therefore, we concluded that there is no reverse causality between the immunophenotype and CRC risk. The results of the reverse MR are presented in Table S1, Supplemental Digital Content 4.

### 3.3. Mediation effect of the immunophenotype on CRC outcomes

To explore the indirect effects of the immunophenotype (including activated CD4 regulatory T cells, CD25++ CD45RA- CD4 nonregulatory T cells (%CD4 + T cells), CD25++ CD45RA- CD4 nonregulatory T cells (%T cells), CD25++ CD8 + T cells (%T cells, and CD64 on CD14 + CD16 + monocytes) on CRC outcomes, we conducted mediation analyses utilizing two-step MR.

**Figure 3.**
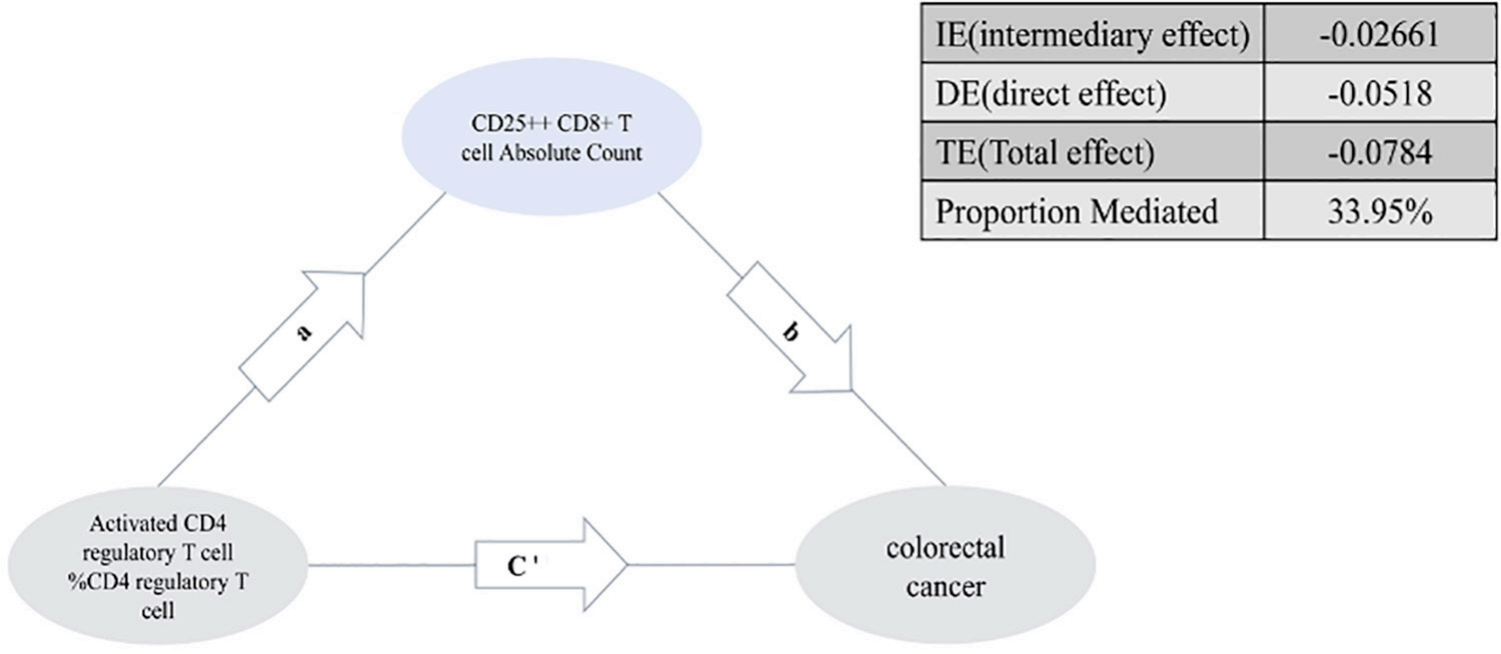
Graphical illustration showing the results of the mediation analysis. Here, the total effect of activated CD4 regulatory T cell %CD4 regulatory T cell on colorectal cancer was estimated as −0.0784; the intermediate effect (a × b) was estimated as −0.0266. Therefore, the direct effect (c’) was equal to −0.0784 + 0.0266 = -0.0518, and the proportion of the mediation effect was calculated as 0.0266/0.0784 = 33.95%.

All the mediation analyses, except one pair of immunophenotypes, identified nonsignificant mediation effects. In this exception, using GWAS data for the immunophenotype (activated CD4 regulatory T cells (%CD4 regulatory T cells)) as the exposure and summary data from ebi-a-GCST90018808 as the outcome, we identified the immunophenotype (CD25++ CD8 + T-cell absolute count) as a mediator linking the immunophenotype with cancer (the IVW method). We identified a positive causal relationship between the percentage of activated CD4 regulatory T cells (%CD4 regulatory T cells) and the absolute count of CD25++ CD8 + T cells (here, activated CD4 regulatory T-cell %CD4 regulatory T-cell is the exposure, and the CD25++ CD8 + T-cell absolute count is the outcome) (*P* = .0017; *a* > 0). Furthermore, we revealed a negative causal effect of the CD25++ CD8 + T-cell absolute count on cancer incidence (with the CD25++ CD8 + T-cell absolute count as the exposure and cancer as the outcome) (*P* = .0108; *b* < 0). As shown in Figure 3, our research demonstrated that the CD25++ CD8 + T-cell absolute count contributed to a 33.9% (95% CI = 9.1%~56.3%) reduction in the risk associated with the activated CD4 + regulatory T-cell percentage of CD4 + regulatory T cells and CRC.

## 4. Discussion

By utilizing a wide range of public genetic data, this study explored the causal relationships between 731 immune cell characteristics via two-sample, reverse, and mediation MR analyses. This study revealed decreased CRC risk with upregulation of activated CD4 regulatory T cells (%CD4 regulatory T cells), CD25++ CD45RA- CD4 nonregulatory T cells (%CD4 + T cells), CD25++ CD45RA- CD4 nonregulatory T cells (%T cells), CD25++ CD8 + T cells (%T cells), and CD64 + CD16 + monocytes. Additionally, interactions between the percentage of activated CD4 regulatory T cells (%CD4 regulatory T cells) and the absolute count of CD25++ CD8 + T cells were observed, collectively influencing CRC risk. To our knowledge, this is the first MR analysis to investigate the causal link between multiple immune phenotypes and CRC.

Studies have revealed that a decreased risk of CRC is associated with an increased proportion of CD64 on CD14 + CD16 + monocytes. A thorough examination of monocyte surface markers identified distinct populations primarily distinguished by CD14 and CD16 expression levels.^[28,29]^ In blood, 3 separate monocyte populations exist: classical CD14++ CD16- monocytes, comprising 85% of circulating monocytes; intermediate CD14 + CD16 + monocytes, representing 5%; and nonclassical CD14 + CD16++ monocytes, making up the remaining 10%. Intermediate and nonclassical monocytes, both derived from classical monocytes, exhibit unique characteristics in terms of migration ability, adhesion molecule expression, cytokine/chemokine profiles, and receptor expression.^[30,31]^ Similar to other diseases, changes in monocyte subset ratios have been noted in various cancer types.^[32–36]^ However, the characteristics of monocyte subsets in cancer patients remain to be clarified.

In this study, a higher frequency of CD25++ CD45RA- nonregulatory CD4 + T cells was correlated with a lower risk of CRC. T regulatory cells (Tregs) were removed from the CD4 + CD25 hi T cells, resulting in the CD25 hiCD4 + non-Treg population (CD25 hiCD127 hi), which is further classified by CD45RA expression. The presence of CD25++ CD45RA- nonregulatory CD4 + T cells is linked to the pathogenesis of allergies, CRO, PSC, and T1D.^[37]^ Currently, no evidence exists showing a relationship between CD25++ CD45RA- nonregulatory CD4 + T cells and CRC development, necessitating further research to confirm any causal relationship. Two-step MR analysis revealed that the CD25++ CD8 + T-cell absolute count was associated with a 33.9% decrease in the risk related to activated CD4 + regulatory T cells (%CD4 + regulatory T cells) and CRC. The HLA DR positivity of CD4 + cells was considered an activation marker.^[16]^ Downregulation of human leukocyte antigen (HLA)-DR expression may impair CD4 + T-cell-mediated antitumor immunity. The findings of this study underscore the critical role of HLA-DR in tumor-immune responses. Previous studies have highlighted HLA-DR’s significant function in the activation and regulation of CD4 + T cell immune responses.^[37]^ CD4 + regulatory T cells (Tregs), characterized by their specific nuclear expression of the transcription factor FoxP3 and the cell surface expression of CD25 and CTLA-4, represent a distinct subset of T cells crucial for preserving immunological self-tolerance and homeostasis. Strategies targeting Tregs, such as reducing their numbers or suppressive activity, are fundamental in eliciting antitumor immune responses or enhancing antimicrobial immunity during chronic infection.^[38]^

FOXP3 + Tregs have been associated with a poor prognosis in several solid tumors, including ovarian,^[39,40]^ pancreatic,^[41]^ and hepatocellular carcinoma.^[42,43]^ However, in CRC, a high density of FOXP3 + Tregs is linked to better survival, differing from findings in other solid tumor types.^[44]^ Moreover, CD25++ CD8 + T cells are associated with IL-2-dependent antitumor responses. The development of antitumor responses dependent on interleukin-2 (IL-2) has focused on targeting the intermediate affinity IL-2 receptor (IL-2R) to activate memory-like CD8 + T cells and natural killer cells, while minimizing the expansion of regulatory T cells (Tregs). In cancer immunotherapy, high-affinity IL-2R biologics are expected to interact with activated tumor antigen-specific CD4 + and CD8 + effector T (Teff) cells that have upregulated CD25, thereby expressing the high-affinity IL-2R.^[45]^ Additionally, IL-2Rα-biased agonists have demonstrated efficacy in enhancing antitumor immunity by stimulating tumor-infiltrating CD25 + CD8 + T cells. Tumor-specific CD8 + T cells (TSTs) co-express high levels of CD25 (IL-2 receptor α-chain) and PD-1, making them more responsive to IL-2Rα-biased agonists.^[46]^ Our research indicates that the activation level of regulatory T cells influences the total number of CD25++ CD8 + T cells, consequently reducing the risk of CRC. These findings enrich our understanding of the interactions among various cell populations in the immune system and offer novel insights for developing immunotherapeutic strategies targeting these cells.

This study, however, has several limitations. First, the number of SNPs for certain immune traits was limited (<5) because of the unavailability of a corresponding GWAS. Although expanding the genetic significance cutoff to 10^−5^, as in other MR analyses ^[16]^, might provide more IVs, this approach risks including weak instruments and increasing horizontal pleiotropy. Additionally, owing to the limited sample size and our decision to maintain strict genetic significance thresholds, we did not adjust for multiple comparisons, which could increase the type I error rate. For the same reason, we used the IVW method as the primary approach because it has the highest statistical power. However, discrepancies between IVW and other pleiotropy-robust MR methods have been observed, warranting further investigation using large-scale GWAS studies. Third, despite conducting multiple sensitivity analyses, the possibility of horizontal pleiotropy cannot be entirely excluded. Finally, while the Mediterranean Sardinian population is widely used in genomics research to identify causal variants in complex diseases such as schizophrenia ^[47]^, it is relatively isolated genetically from the mainland European population. In theory, if the exposure and outcome populations differ substantially, the MR results could be biased owing to pleiotropy, weak instruments, or confounding by ancestry. However, we addressed these potential issues by performing multiple sensitivity analyses, including alternative MR methods, leave-one-out analysis, heterogeneity testing, and the exclusion of SNPs with *F*-statistics <10, along with validation steps using both discovery and replication datasets to ensure robust causal inference. Certainly, further genetic profiling using a diverse range of European populations is also recommended.

## 5. Conclusion

The IVW method revealed that 5 immunophenotypes may be risk factors for CRC. However, other complementary methods yielded insignificant results. Further investigation of these immunophenotypes as risk factors and thus potential therapeutic targets for CRC is strongly recommended.

## Acknowledgments

This study was supported by funds from the Jilin Provincial Science and Technology Project (2019SCZ007).

## Author contributions

**Formal analysis:** Yi Zhang, Bingyuan Fei.

**Data curation:** Bingyuan Fei.

**Conceptualization:** Xuedong Fang, Suyan Tian.

**Supervision:** Xuedong Fang.

**Writing – original draft:** Yi Zhang, Suyan Tian.

**Writing – review & editing:** Xuedong Fang, Suyan Tian.








