# Supplementary figures and images for: To unveil the causal relationship between immunophenotypes and colorectal cancer using two-sample bidirectional Mendelian randomization and mediation analyses
Source: Medicine (Baltimore). 2026 Jul 10;105(28):e49769. doi: 10.1097/MD.0000000000049769 (PMC13363119; doi:10.1097/MD.0000000000049769)

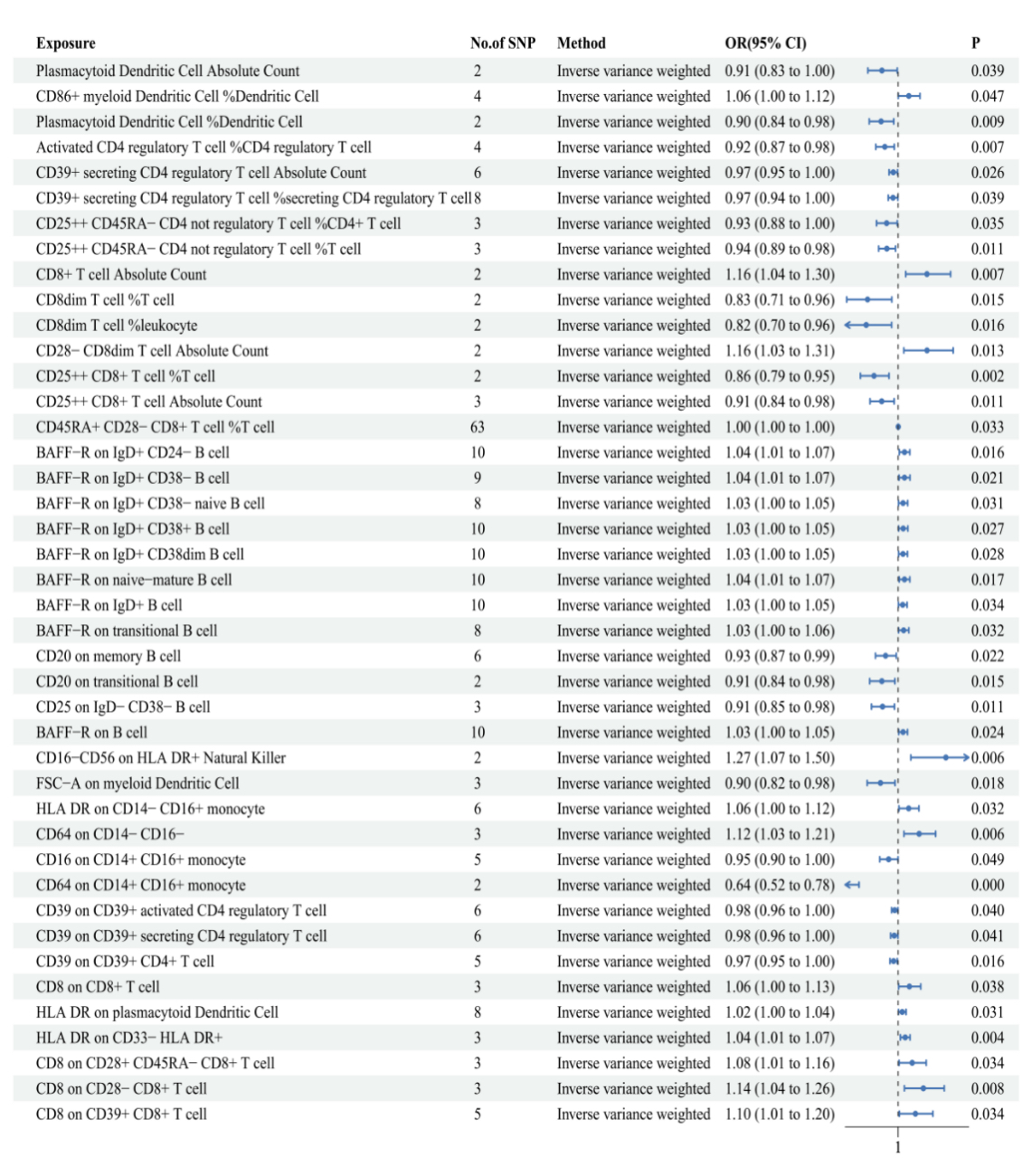

Supplement: Supplementary file 1 [file medi-105-e49769-s001.jpg]

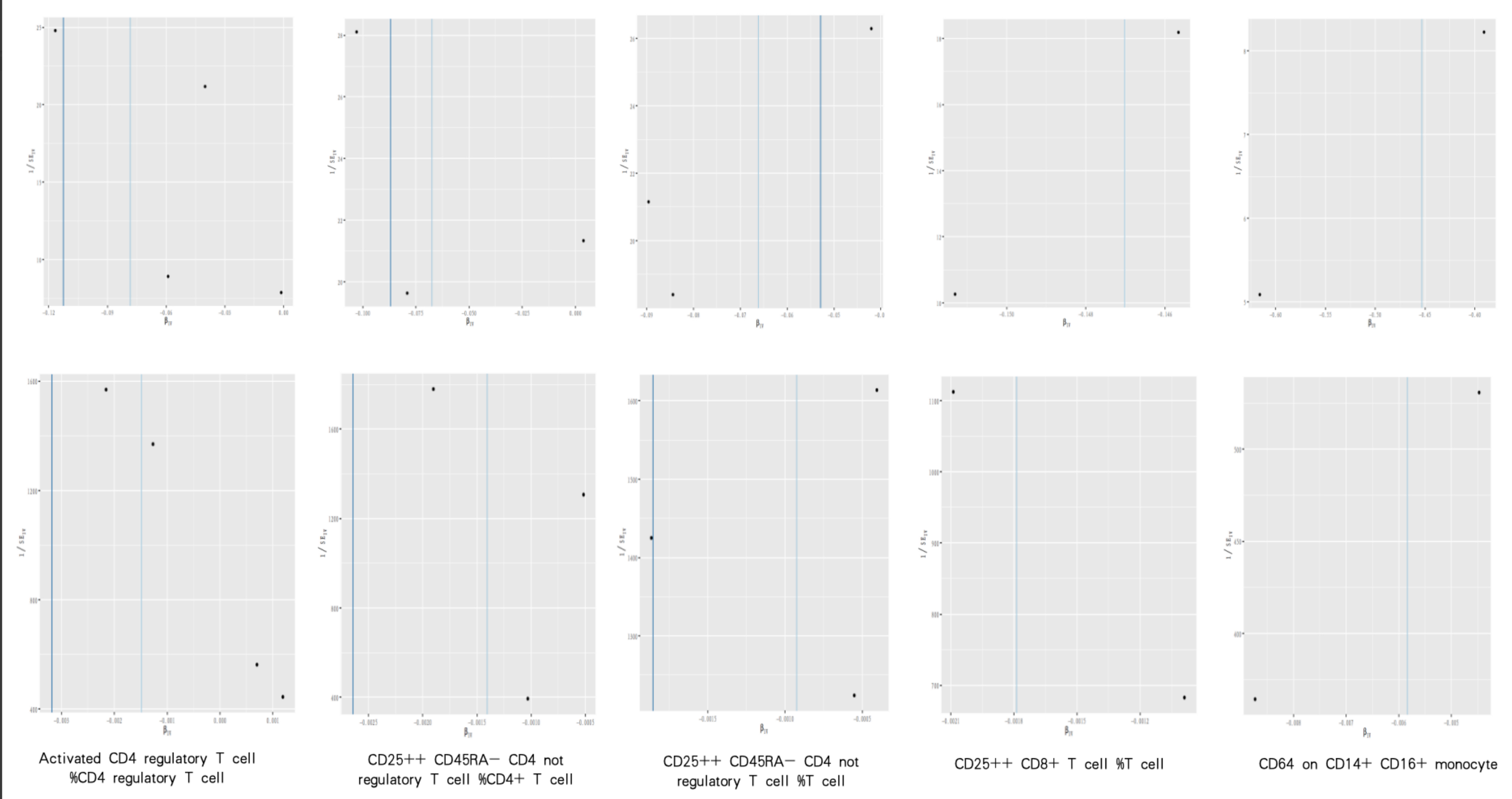

Supplement: Supplementary file 2 [file medi-105-e49769-s002.jpg]

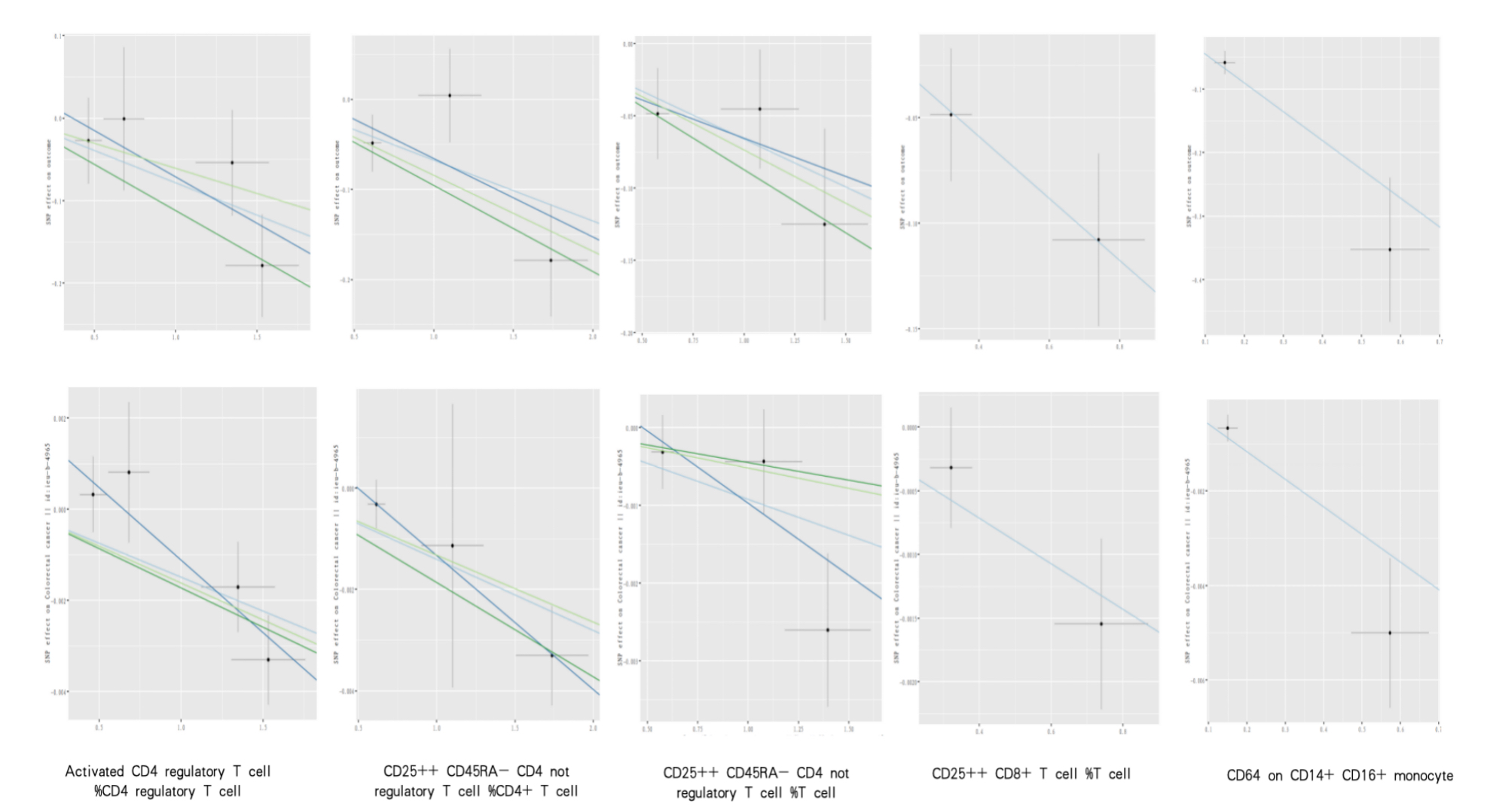

Supplement: Supplementary file 3 [file medi-105-e49769-s003.jpg]
