# Supplementary material for: To unveil the causal relationship between immunophenotypes and colorectal cancer using two-sample bidirectional Mendelian randomization and mediation analyses
Source: Medicine (Baltimore). 2026 Jul 10;105(28):e49769. doi: 10.1097/MD.0000000000049769 (PMC13363119; doi:10.1097/MD.0000000000049769)
Supplement: Supplementary file 4 [file medi-105-e49769-s004.docx]

**Table S1.** Results of the casual effect of colorectal cancer on immune traits (results of reverse Mendelian randomization)

| Exposure | Outcomes | No. of SNP | Method | OR | OR_lci95 | OR_uci95 | P |
| --- | --- | --- | --- | --- | --- | --- | --- |
| Colorectal Cancer | Activated CD4 regulatory T cell %CD4 regulatory T cell | 25 | Inverse variance weighted | 1.074 | 0.943 | 1.224 | 0.281 |
|  |  |  | MR Egger | 1.184 | 0.689 | 2.034 | 0.547 |
|  |  |  | Weighted median | 1.154 | 0.983 | 1.354 | 0.081 |
|  |  |  | Weighted mode | 1.123 | 0.906 | 1.393 | 0.298 |
|  | CD25++ CD45RA- CD4 not regulatory T cell %CD4+ T cell | 25 | Inverse variance weighted | 1.074 | 0.962 | 1.200 | 0.206 |
|  |  |  | MR Egger | 0.962 | 0.613 | 1.509 | 0.866 |
|  |  |  | Weighted median | 1.114 | 0.952 | 1.304 | 0.179 |
|  |  |  | Weighted mode | 1.166 | 0.898 | 1.514 | 0.261 |
|  | CD25++ CD45RA- CD4 not regulatory T cell %T cell | 25 | Inverse variance weighted | 1.054 | 0.944 | 1.177 | 0.352 |
|  |  |  | MR Egger | 0.941 | 0.600 | 1.475 | 0.792 |
|  |  |  | Weighted median | 1.082 | 0.923 | 1.268 | 0.329 |
|  |  |  | Weighted mode | 1.131 | 0.874 | 1.464 | 0.359 |
|  | CD25++ CD8+ T cell %T cell | 25 | Inverse variance weighted | 0.996 | 0.882 | 1.125 | 0.951 |
|  |  |  | MR Egger | 0.936 | 0.565 | 1.549 | 0.798 |
|  |  |  | Weighted median | 0.990 | 0.843 | 1.163 | 0.907 |
|  |  |  | Weighted mode | 0.993 | 0.770 | 1.282 | 0.959 |
|  | CD64 on CD14+ CD16+ monocyte | 25 | Inverse variance weighted | 0.938 | 0.842 | 1.044 | 0.243 |
|  |  |  | MR Egger | 0.793 | 0.511 | 1.229 | 0.310 |
|  |  |  | Weighted median | 0.946 | 0.820 | 1.092 | 0.448 |
|  |  |  | Weighted mode | 0.933 | 0.757 | 1.150 | 0.521 |
